# Operationalizing Stakeholder Engagement Through the Stakeholder-Centric Engagement Charter (SCEC)

**DOI:** 10.1007/s11606-021-07029-4

**Published:** 2022-03-29

**Authors:** Jenny Martínez, Catherine Verrier Piersol, Kenneth Lucas, Natalie E. Leland

**Affiliations:** 1 Department of Occupational Therapy, Jefferson College of Rehabilitation Sciences, Thomas Jefferson University, Philadelphia, PA USA; 2 Department of Occupational Therapy, School of Health and Rehabilitation Sciences, University of Pittsburgh, Pittsburgh, PA USA; 3 Advisory Committee Member, Philadelphia, PA USA

## Abstract

There is a need for clear strategies and procedures to operationalize stakeholder engagement in research studies. Clear guidelines that promote shared leadership among study investigators and research stakeholders are important for inclusive and sustainable partnerships. Such guidelines may take the form of a governance charter and can be a means for encouraging the participation and inclusion of stakeholders who may have little to no experience with research or are otherwise underrepresented in research. This perspective article presents the Stakeholder-Centric Engagement Charter (SCEC), one effort to operationalize a stakeholder engagement approach between researchers and an advisory committee as guided by the Patient-Centered Outcomes Research Institute’s (PCORI) Research Engagement Principles (i.e., reciprocal relationships, partnerships, co-learning, transparency-honesty-trust). Building on the SCEC can help future investigators develop a study-specific, dynamic, governance document outlining advisory committee and research team preferences in areas such as role expectations, study governance, and decision-making procedures.

**Supplementary Information:**

The online version contains supplementary material available at 10.1007/s11606-021-07029-4.

## INTRODUCTION

Systematically and purposefully engaging healthcare stakeholders (e.g., patients, caregivers, practitioners, policy makers, and payers) in research activities is one strategy for producing findings that are actionable, timely, and in direct alignment with stakeholder priorities or needs.^1–3^ To achieve this goal, research teams must formalize engagement practices as part of the ongoing study operations in order to systematically champion transparency and power sharing. This intentional approach to engagement is essential for collaborating with those most vulnerable such as individuals with little to no research experience, limited English proficiency, and distrust in research or the healthcare system, and/or those experiencing healthcare disparities.^1,4–7^

## OPERATIONALIZING STAKEHOLDER ENGAGEMENT IN RESEARCH

Stakeholder engagement in research often manifests as governance structures such as steering committees, advisory boards, or advisory committees.^2,8,9^ Stakeholder roles and responsibilities vary in complexity and depth across studies, ranging from information exchange to consultation or shared decision-making.^1,2,7,10,11^ Studies evaluating the implementation of governance structures from stakeholders’ perspectives report (a) restricted power to influence the research agenda, (b) minimal comprehension about the study, (c) disempowered relationships with researchers, (d) insufficient or absent incentives for participation given competing demands on stakeholder time, and (e) inadequate guidelines for governance, collaboration, or decision-making.^1,7,10–12^ Thus, there is an urgent need for research governance structures that foster meaningful stakeholder engagement.

A charter is one approach to establishing guidelines, delineating roles, and clarifying policies for study governance structures. A charter can promote researcher-stakeholder partnerships that are authentic, inclusive, and sustainable by integrating best practices for engagement and including stakeholders in its development.^13,14^ The Engagement Rubric developed by the Patient-Centered Outcomes Research Institute (PCORI) is one such stakeholder engagement framework. This Engagement Rubric introduces the Patient-Centered Outcomes Research Engagement Principles: reciprocal relationships, partnerships, co-learning, transparency-honesty-trust.^15^ Further, recent research offers information about emerging engagement challenges, potential strategies to address such challenges, and lessons learned—yet there is still a need for specific methods and procedures for engaging stakeholders.^3,11,12,16,17^

To address this gap, we describe our process for collaborating with our stakeholder advisory committee to develop a study charter, which we call Stakeholder-Centric Engagement Charter (SCEC). The SCEC operationalized engagement within a PCORI-funded large pragmatic trial (ClinicalTrials.gov Identifier: IHS-1608-35732). We share our experiences and the SCEC to help future investigators customize a study charter or governance plan in collaboration with their stakeholders that best meets their study’s needs.

## DEVELOPING THE STAKEHOLDER-CENTRIC ENGAGEMENT CHARTER

The SCEC was developed in collaboration between the *research team* and *advisory committee*. The research team (clinical and academic principal- and co-investigators) leveraged longstanding professional networks to convene an 18-member *advisory committee* that guided the development of the research application to PCORI. The advisory committee included representation from stakeholder communities such as patient advocates, caregivers, practitioners, policy makers, and health system leadership.^2,18^ Further details about our advisory committee collaborators have been described previously.^13^

Upon notice of funding, we convened an in-person study kickoff meeting that brought the advisory committee and the research team together. The objective of the kickoff meeting was to develop clear working guidelines for stakeholder engagement and has been described elsewhere.^13^ Given scarce literature on how to operationalize stakeholder engagement, we relied on emerging funder guidance (i.e., PCORI’s Research Engagement Principles)^15^ and commonly used resources for developing governance structures or facilitating committee business (i.e., Robert’s Rules of Order, University of Kansas’ Community Tool Box). ^19,20^ An initial outline of the SCEC was developed by the research team prior to the kickoff meeting with special attention to calls for valuing stakeholders’ time and streamlining burden in research partnerships.^3,17,21^ The draft charter broadly reflected PCORI’s Research Engagement Principles including (a) expectations for participation, meeting procedures, and resignation/removal/succession from the committee (reciprocal relationships); (b) expectations for compensation, recognizing the voluntary nature of participation and the need to support members through hardships that may impact participation (partnerships); (c) a commitment to learning from members’ primary perspectives (co-learning); and (d) procedures for conflict resolution (transparency-honesty-trust).

The advisory committee and research team discussed stakeholder engagement and the SCEC outline during the kickoff meeting.^13,15,17^ This process was integral to jointly identifying reasonable expectations and forming a culture of shared leadership. Next, the advisory committee and research team operationalized the outline into actionable policies and shared expectations. This process was anchored by open discussions in small groups to (a) elicit advisory committee member’s thoughts and recommendations, (b) develop a study governance structure that met funder and advisory committee expectations, and (c) synthesize advisory committee recommendations to ensure translation into clear, actionable procedures.

To promote equity in study leadership, we requested targeted review of the SCEC by advisory committee members with little experience in research and those representing patient or caregiver perspectives. In recognition of their time, compensation was provided to reviewers. For members limited by technology literary or access, review occurred via phone calls, texts, and/or printed materials. Reviews centered on how well the SCEC captured advisory committee member feedback and was accessible to non-researchers. Next, the SCEC was sent to the entire advisory committee for final review and approval. All members unanimously approved the final version of the SCEC in the form of a signature. Appendix presents the SCEC in its entirety.

## ENHANCING THE STUDY THROUGH THE STAKEHOLDER-CENTRIC ENGAGEMENT CHARTER

The SCEC was integral to our study in the following ways: (a) strengthening advisory committee enthusiasm and investment in the study; (b) clarifying expectations for the research team and advisory committee; (c) operationalizing stakeholder engagement; and (d) preventing and resolving conflict. We describe each area in more detail below.

### Strengthening Advisory Committee Enthusiasm and Investment in the Study

Advisory committee members overwhelmingly shared positive feedback about their participation. This data was gathered via a separate biannual survey to elicit ongoing feedback from advisory committee members about their experiences as research collaborators (results published previously).^13^ The research team monitored the survey for responses indicating the need to revise the study procedures outlined in the SCEC. Instead, responses reflected advisory committee members’ positive opinions and investment in the study. One advisory committee member stated:

The research team does an excellent job keeping the advisory committee in the loop. The team is very organized and the monthly meetings and updates run very smoothly. I look forward to hearing more once the trainings and caregiver feedback is received. The team is very impressive. Thank you.

### Clarifying Partnership Expectations

The SCEC clarified fair financial compensation for stakeholder partners, participation expectations, (e.g., attendance requirements, compensation, participation schedule), and reasonable requests for time. For example, in recognition of the value of each advisory committee member, compensation for advisory committee members was commensurate with the stipend provided to any other project consultant, regardless of degree or title. We also created a monthly meeting schedule with multiple meeting times each month to accommodate stakeholders’ busy schedules and varying time zones. The advisory committee suggested and unanimously approved a recommendation that no compensation be provided to members who were more than 15 minutes late to a project meeting. The research team developed a meeting schedule for the coming year and disseminated meeting agendas at least 2 weeks before each monthly meeting to offer transparency. These procedures yielded considerable success and resulted in an average attendance of 16 out of 18 advisory committee members on any given month.

### Operationalizing Stakeholder Engagement

The SCEC was essential to operationalizing engagement procedures such as onboarding members and facilitating monthly meetings. New research team and advisory committee members met one-on-one with a research team member to review our collective values and discuss charter domains before signing. Furthermore, the SCEC identified predictable, organized, and systematic procedures for our monthly meetings. For example, we identified timelines for scheduling meetings, developing agendas, sharing meeting materials, posting meeting minutes for review or approval, and participation in study discussions. We clarified communication procedures that utilized technology at no cost to advisory committee members, such as Zoom for videoconferencing, Basecamp for project management, and REDCap to distribute a biannual stakeholder engagement survey.

### Preventing and Resolving Conflict

The SCEC helped prevent conflict by promoting shared governance and operating procedures that had advisory committee input and buy-in. For example, we identified potential barriers to advisory committee participation (e.g., limited access to technology or time constraints) and jointly developed creative solutions such as establishing methods for providing technology or caregiving support, communicating online, and adapting meeting schedules to meet members’ busy schedules. We also budgeted funds to financially support advisory committee member participation and mitigate barriers to engagement (e.g., need for cell phone or webcam access) while maintaining the member’s confidentiality.

Furthermore, the SCEC outlined procedures to successfully resolve disagreements. For example, in one occasion when one member unintentionally made a negative comment about the knowledge of another member, the study principal investigators followed the SCEC procedures to provide a confidential space for those involved to discuss the event. The study principal investigators also used this opportunity to reinforce the value of the advisory committee’s expertise, make amends, and reinforce our collective values. This approach was successful and both members remained active advisory committee participants throughout the study.

In another situation, the SCEC guided the process of providing feedback to three inaugural advisory committee members who were not participating as expected. Following a review of the SCEC’s expectations for participation, the advisory committee members opted to resign from the advisory committee given their busy schedules. Both members expressed ongoing commitment to the project and identified successors who remained active participants throughout the rest of the study. These were the only changes across the advisory committee membership’s 18-person membership.

## CONCLUSION

Engaging stakeholders in ongoing research is a valuable strategy for enhancing research transparency, applicability, and trustworthiness. Although previous literature offers lessons learned and potential guidelines for engagement, there is a need for peer-reviewed evidence that builds on this foundational work to provide actionable and specific strategies for operationalizing researcher-stakeholder partnerships.^1,3,5,11,16,22^ Given the diversity of stakeholders’ perspectives, experiences, familiarity with research, and trust in healthcare, it is especially important that investigators understand best practices for leading and fostering stakeholder engagement that is equitable, sustainable, and transparent. Furthermore, such research should readily share power with non-researchers and historically underrepresented individuals within the research enterprise. In this way, scientists can enhance the likelihood of producing research that is timely and more easily implementable and addresses longstanding health outcomes perpetuated by systemic inequity, unequal access to resources, and marginalization of vulnerable populations.^1^

The process of collaboratively developing the SCEC facilitated the buy-in from our advisory committee who played an active role in developing the SCEC. Our continued adherence to the SCEC throughout the study further demonstrated our commitment to inclusive and transparent practices and facilitated trust between the research team and advisory committee. Although the charter we present here was developed at a time when little information was available on conducting stakeholder engagement research, our approach can serve as a guiding framework and resource for investigators who are working to operationalize stakeholder engagement in their investigations. For example, investigators may use the SCEC as a framework for a guided discussion. Such discussions can lead to the development of a study-specific, dynamic, governance document outlining advisory committee and research team preferences in areas such as role expectations, study governance, and decision-making procedures.

As stakeholder engagement literature continues to grow and additional resources become available, future studies could analyze the growing number of materials, including on PCORI’s newly available *Engagement Tool and Resource Repository*, to identify common practices for collaboration.^23^ In addition, areas for further investigation include stakeholder implementation of governance guidelines like the SCEC, strategies for enhancing shared governance in research, accessibility of documents to non-researchers, evaluation of engagement approaches, and additional ways to collaborate among a range of stakeholders to advance the science of stakeholder engagement.^3,11,16,23^

## Supplementary Information

ESM 1 (PDF 164 kb)

## Declarations

### Disclaimer

The statements in this publication are solely the responsibility of the authors and do not necessarily represent the views of the Patient-Centered Outcomes Research Institute (PCORI), its Board of Governors, or Methodology Committee.
