# Supplementary material for: Operationalizing Stakeholder Engagement Through the Stakeholder-Centric Engagement Charter (SCEC)
Source: J Gen Intern Med. 2022 Mar 29;37(Suppl 1):105–8. doi: 10.1007/s11606-021-07029-4 (PMC8994015; doi:10.1007/s11606-021-07029-4)
Supplement: Supplementary file 1 — (PDF 164 kb) [file 11606_2021_7029_MOESM1_ESM.pdf]

Appendix A

The Stakeholder-centric Engagement Charter (SCEC)

Project title: [Insert study title here]

Advisory Committee Participation Overview

One-hour advisory committee meetings will be held monthly during the study funding period [specify period here] and electronic communication will be used as needed. Meetings will occur via videoconference and a schedule of meetings along with the call-in information will be provided to advisory committee members in advance. A standing time and date will be discussed during the In-person Kickoff Meeting at the beginning of this study and approved by meeting attendees.

In the case that the meeting schedule is adjusted (e.g., a month is skipped or other changes), the advisory committee will be notified as soon as possible.

[Identify investigators and their roles in the study] will facilitate the monthly committee meetings. [Identify investigators and their roles in the study] will (a) serve as a liaison between the research team and the advisory committee, managing all communications between them; (b) be available to consult with the advisory committee and principal investigators regarding the concerns of the committee; (c) preside over advisory committee meetings when the study PIs are unable to do so; (d) approve information sent to the committee, meeting agendas for the advisory committee and meeting schedules to assure that there is sufficient time for discussion of all agenda items.

Membership in this advisory committee is public information. Thus as a member of the advisory committee you are welcome to disclose your participation. However, all advisory committee meeting proceedings are closed and the information discussed herein confidential. The Co-PIs may

from time to time invite other advisors of team members to attend committee meetings whenever deemed appropriate. Advisory committee members who wish to share information with non-committee members or extend an invitation to participate in monthly meetings must obtain the approval of both Co-PIs in writing (e.g., via email).

Agenda Items for Committee Meetings

[Identify investigators and their roles in the study] will establish a structure for the agenda (e.g., recruitment status, methodology feedback, dissemination activities, etc.). The scientific project manager will then develop the monthly agenda for each meeting following this structure. Two weeks prior to the monthly meeting, the will scientific project manager email all involved parties to solicit any agenda items. The final monthly agenda will include (a) the initial items suggested by the Co-PIs and stakeholder engagement lead; (b) any additional items suggested by the Co-PIs, stakeholder engagement lead, and research team; (c) additional items suggested by the advisory committee members; (d) action items agreed upon in previous meetings whose status must be discussed.

Meeting agendas and previous meeting minutes will be available to all advisory committee members and archived on Basecamp.com, a website platform that allows for sharing of documents and team communication. A detailed agenda and, to the extent feasible, supporting documents and proposed resolutions will be provided to the advisory committee approximately three days prior to each meeting. Advisory committee members should review these materials in advance of the meeting.

During the monthly advisory committee calls, as new issues arise or priority topics are identified, they will be documented in the meeting notes and prioritized for the following month's agenda. More specifically, at the end of the call, we will summarize any issues that came up and determine if additional items need to be added to the next month's agenda in collaborating with advisory committee members.

[Identify investigators and their roles in the study] will be responsible for recording minutes during each meeting as well as attendance. Following each meeting, minutes will be uploaded to the advisory committee's Basecamp.com page so that they are easily accessible by all committee members. Meeting minutes will be completed and uploaded to the advisory committee's Basecamp.com page within 24 hours.

Following each monthly advisory committee call, [Identify investigators and their roles in the study] will post a follow-up topic or facilitated discussion on the Discussion Forum of the advisory committee's Basecamp.com page. Advisory committee members will be able to respond with any additional thoughts following the scheduled call. In addition, any committee members who were absent from the monthly call will be required to review the posted meeting minutes and provide their response on the team's Discussion Forum within 2 weeks of the missed meeting.

Advisory Committee Member Compensation

Compensation for meeting participation for advisory committee members is [Insert amount here]. Prior to the start of each meeting, [Identify investigators and their roles in the study] will

record attendance and use this record to disseminate payment to present committee members. Please note that compensation for participation cannot be provided to an advisory committee member who arrives more than 15 minutes late or cannot otherwise attend. Any advisory committee member who is unable to attend is required to review the minutes for that meeting and respond to action items as indicated.

Advisory Committee Orientation and Education

Advisory committee members will receive a copy of participation expectations and be oriented to them during the initial In-person Kickoff Meeting for this study. Modifications to this agreement shall be discussed at that time. New members will review this charter upon joining the study. Following the discussion, committee members will be asked to indicate their agreement and understanding of the material discussed via their signature. Any additional questions following review of the charter should be directed to [Identify investigators and their roles in the study] throughout the duration of the study.

Advisory Committee Expectations for Participation

Advisory committee members are expected to attend all scheduled monthly meetings. These meetings are designed to provide the research team an opportunity to discuss study progress and request input from the advisory committee. A committee member may not miss more than 3 meetings per year. If an absence is expected, committee members are required to provide at least a

48-hour notice via email to [Identify investigators and their roles in the study]. Any emergencies that impact attendance will be managed on a case by case basis. In addition, arriving more than 15 minutes late to a meeting will count as a missed call.

Any advisory committee member who is absent from a call will be asked to review the posted minutes and provide their response and thoughts on the Discussion Forum of the advisory committee's Basecamp.com page within 2 weeks of the missed meeting.

The guiding principles for this study, and thus, this advisory committee include:

- Reciprocal relationships – clearly and collaboratively defined roles and decision-making authority
- Co-learning – learning from each other
- Partnerships – value the time and contributions of our stakeholders
- Transparency, honesty, and trust— inclusive information-sharing and information sharing
- Collegiality– united in a common purpose and respecting each other's abilities to work toward it
- Power sharing– share power within the team
- Confidentiality– protect all information shared

All advisory committee meeting proceedings are closed and the information discussed herein confidential. In addition, all emails, materials, and communications shared with advisory committee members should be considered private and confidential unless otherwise specified. In addition, given the study's use of blinding, any knowledge of allocation of a treatment facility must also be kept confidential to maintain scientific integrity and rigor. For advisory committee members who

may interact with visual resource materials or training records at their day to day work, this information should be kept confidential and not be discussed at committee meetings.

All research team members, including the advisory committee are expected to adhere to these principles at all times. Through these actions, we hope to create an environment where all advisory committee members feel equally valued, heard, and appreciated. To strengthen these efforts, all members of the research team and advisory committee will address each other using their first names, excluding titles (e.g., Dr.) and degrees or certifications (e.g., PhD, MD, etc.) in all communications. Though this effort, we hope to create an egalitarian environment where all team members feel comfortable and are willing to contribute their valuable expertise with the entirety of the research team.

Research Team Expectations

The scientific and clinical team will also abide by the expectations presented in the section titled Advisory Committee Orientation and Education. In addition, the scientific and clinical teams are committed to presenting all study-related information in a clear, accessible, and jargon-free manner that is easily understood by the wide range of stakeholders on our advisory committee (e.g., patient, clinicians, researchers, clinicians, healthy systems, etc.).

Any suggestions from advisory committee members are welcome regarding ways for the scientific and clinical team to improve study communications. In addition, if a committee member would like to further discuss a document or review material presented in previous meeting, [Identify

investigators and their roles in the study] will serve as a first point of contact. If further input from team members is needed, she will liaison as necessary to ensure that the advisory committee member's questions have been fully answered.

Advisory Committee Composition and Membership

Advisory committee members were invited to participate at the onset of proposal development given their expertise in a wide range of areas and practice relevant to the study. The primary perspective that each advisory committee member represents in this study was jointly determined at the time of invitation and is reflected on the accompanying team roster. Although membership across multiple stakeholder groups (e.g., patient, clinicians, researchers, clinicians, healthy systems, etc.) is possible and informs advisory committee member input, we request that committee members share from the primary perspective which they represent on our study. This information can be found on the Advisory Committee Members document which will also be uploaded to Basecamp.com for easy access.

Affiliation with a participating university or clinical partner is not a prerequisite for participation. A status in change of employment or position shall not impact the role of the committee member or their membership on the study advisory committee.

Conflict Resolution Procedures

The advisory committee's assistance and oversight will be requested to resolve any conflicts that may arise between the PIs or the scientific and clinical research team. In addition, the advisory committee's guidance will also be requested for major study-related concerns such as data collection and interpretation processes.

In the case that the advisory committee cannot come to a consensus on a particular issue, we will discuss the issue and achieve consensus. If the group or team is not able to achieve consensus on the topic, all advisory committee and research team members will vote on the recommendations and the majority vote will prevail.

[Identify investigators and their roles in the study] will also serve as a first point of contact if any advisory committee has any concern regarding, or experiences conflict and would like to share this concern confidentially with the team. [Identify investigators and their roles in the study] will liaison between the Co-PIs and the advisory committee member to establish a plan for addressing the concern raised.

Resignation from the Advisory Committee

Any advisory committee member may resign at any time by giving notice in writing or by electronic transmission to [Identify investigators and their roles in the study]. Such resignation shall

take effect upon the effective date stated by the advisory committee member. The acceptance of such resignation by the research team shall not be necessary to make it effective.

If a committee member should experience any hardship that has the potential to impact their participation in this study, they should feel free to contact [Identify investigators and their roles in the study] to create a plan for continued participation. In addition, if a committee member needs technological support or access to the technology required to participate in the study monthly calls, they should feel free to contact [Identify investigators and their roles in the study] to obtain confidential support.

Removal from the Advisory Committee

Any advisory committee member may be removed from membership under the following circumstances:

- Repeated unresponsiveness (more than 3 email attempts) to study-related emails requesting action or feedback
- Being absent from more than 3 advisory committee meetings per year
- Not reviewing meeting minutes and providing their thoughts on the Discussion Forum of Basecamp.com following a meeting absence
- Not providing previous notice regarding any absence through email to the research team as specified in the prior section titled Advisory Committee Expectations for Participation
- Creating and/or contributing to an environment contrary to that outlined in the prior section titled Advisory Committee Expectations for Participation

- Not following privacy and confidentiality guidelines

Prior to making a final decision, the advisory committee member will be contacted by a study Co-PI to discuss the situation with the committee member. If the issue is not resolved, both study Co-PIs will discuss the matter and must agree before an advisory committee member is removed and will notify the member in writing (e.g. email).

Successors to the Advisory Committee

In the case that an advisory committee member resigns or is removed, the advisory committee will be asked to help identify a successor that represents the perspective of the previous committee member.

Dissemination Activities

Advisory committee members may be approached by the research team to collaborate on a peer-reviewed manuscript, article, or presentation related to this study. Such opportunities may also be discussed during committee monthly meetings to gauge member interest. Prior experience in any of these dissemination activities is not required. In addition, any committee members who would like to participate in dissemination activities should feel free to voice their interest in monthly meetings of via email to [Identify investigators and their roles in the study] who will maintain a list of interested members.

Any advisory committee member who is interested in leading or participating a dissemination activity within their organization or elsewhere must notify [Identify investigators and their roles in the study] and submit a completed request form along with their request. Blank versions of the Manuscript Proposal Form and the Conference Abstract Proposal Form will be available on Basecamp.com. A blank version of the Other Dissemination Proposal Form will be available for other dissemination requests (e.g., advocacy, etc.). If an advisory committee member would like assistance completing the form, [Identify investigators and their roles in the study] will work with the committee member to complete the form.

Once a completed proposal form has been submitted to [Identify investigators and their roles in the study], they will present it at the following scientific meeting where the research team will evaluate it for appropriateness and its alignment with the study's confidentiality needs. Following this evaluation, [Identify investigators and their roles in the study] will relay this decision and any

next steps to the interested advisory committee member. In some cases, a research team member may oversee the representation of this study in the requested dissemination activity.

Agreement

My signature on this document reflects my understanding of these guidelines and commitment to abiding by them. A copy of this document will also be provided and hosted on Basecamp.com.

Date: [Date document]

______________________________________ ______________________________________

______________________________________ ______________________________________

______________________________________ ______________________________________
